# Towards Automated Classification of Zooplankton Using Combination of Laser Spectral Techniques and Advanced Chemometrics

**DOI:** 10.3390/s22218234

**Published:** 2022-10-27

**Authors:** Nikolai I. Sushkov, Gábor Galbács, Patrick Janovszky, Nikolay V. Lobus, Timur A. Labutin

**Affiliations:** 1 Department of Chemistry, Lomonosov Moscow State University, 119234 Moscow, Russia; 2 Department of Inorganic and Analytical Chemistry, Faculty of Science and Informatics, University of Szeged, 6720 Szeged, Hungary; 3 Timiryazev Institute of Plant Physiology, Russian Academy of Sciences, 127276 Moscow, Russia

**Keywords:** zooplankton, LIBS, Raman, chemometrics, classification, PCA, NMF, ComDim

## Abstract

Zooplankton identification has been the subject of many studies. They are mainly based on the analysis of photographs (computer vision). However, spectroscopic techniques can be a good alternative due to the valuable additional information that they provide. We tested the performance of several chemometric techniques (principal component analysis (PCA), non-negative matrix factorisation (NMF), and common dimensions and specific weights analysis (CCSWA of ComDim)) for the unsupervised classification of zooplankton species based on their spectra. The spectra were obtained using laser-induced breakdown spectroscopy (LIBS) and Raman spectroscopy. It was convenient to assess the discriminative power in terms of silhouette metrics (*Sil*). The LIBS data were substantially more useful for the task than the Raman spectra, although the best results were achieved for the combined LIBS + Raman dataset (best *Sil* = 0.67). Although NMF (*Sil* = 0.63) and ComDim (*Sil* = 0.39) gave interesting information in the loadings, PCA was generally enough for the discrimination based on the score graphs. The distinguishing between *Calanoida* and *Euphausiacea* crustaceans and *Limacina helicina* sea snails has proved possible, probably because of their different mineral compositions. Conversely, arrow worms (*Parasagitta elegans*) usually fell into the same class with *Calanoida* despite the differences in their Raman spectra.

## 1. Introduction

Analytical chemistry, especially in its applied branches, is largely related to decision making. For example, analytical measurements in environmental studies are needed to decide whether a sampled material is of interest for further work due to the presence of a specific marker or due to a high content of a hazardous substance [1]. Subsequent remedial or handling actions depend on the quantitative result of the measurement converted into categorial variables. The most evident example is the unsupervised classification [2] of unknown samples based on their analytical signals.

One of the fields where classification is extensively used is the analysis of biological materials—from crops and food to bacteria and human tissues [3,4,5]. Classification can be based on different types of information. The most evident way is to use optical images with a proper mathematical treatment (computer vision). This idea has been fruitful, e.g., in zooplankton identification, either in a supervised [6,7] or an unsupervised fashion [8,9,10,11]. However, spectroscopy can be used for individual tissues when simple or even hyperspectral imaging is not an ultimate solution. Rapid, sample-sparing, and information-rich analytical methods, such as laser-induced breakdown spectroscopy (LIBS) [12,13,14,15,16], Raman [17,18], and near-infrared (NIR) spectroscopies [19,20], supported by numerous mathematical techniques of multivariate data analysis, have come into question during the past two decades. Many popular algorithms, such as discriminant analysis based on projection on latent structures (PLS-DA), discriminant function analysis (DFA), the k-nearest neighbour method (kNN), soft independent modelling of class analogies (SIMCA), support vector machine (SVM), classification trees (CTs), random forest (RF), and artificial neural networks (ANNs) [2,21,22,23,24,25,26,27,28,29,30], are supervised ones and thus require a training set of samples. Of course, this is not always possible, especially when there is no idea about how the classes should be defined.

The most common unsupervised technique is the principal component analysis (PCA) [31]. There are many other algorithms of this kind, e.g., non-negative matrix factorisation (NMF) [32,33] or independent component analysis (ICA) [34]. The common feature of these techniques is that the user obtains a set of scores which can be used for classification. NMF does not require its latent vectors to be orthogonal, which is a benefit for mixtures sharing some signals, but the solution is not unique. In other words, decomposition results in an infinite set of solutions due to the so-called rotational ambiguity [35,36]. On the other hand, the solution is always non-negative, which is convenient when working with many spectroscopic techniques (such as LIBS and Raman spectroscopy) where signals cannot be negative; NMF thus gives only those solutions that can be physically valid.

The independent component analysis (ICA) technique was initially used for blind source separation [37] and now finds many successful applications in fluorescence, nuclear magnetic resonance (NMR), infrared (IR) and Raman spectroscopy, chromatography and electrochemistry [34,38,39]. In addition to the non-orthogonality of the components, which brings the decomposition closer to physical reality [40,41,42] (the overlapping of signals of different compounds means that the spectra are not completely different and thus cannot be orthogonal—this is important for complex mixtures of natural compounds), ICA assumes that the components are non-Gaussian [40]. Thus, ICA not only tries to minimize correlation between the components, it also maximizes the deviation from normal distribution to obtain “independent” components.

Chemometrics can be applied to the detection of adulterations in the food industry, the detection and discrimination of pathogenic microorganisms or chemical warfare agents, the discrimination between diseased and healthy organisms or tissues, quality control, etc. [5]. The advantages of rapid spectroscopic techniques such as LIBS or Raman spectroscopy are the possible automations, the speeding up and scaling of analytical procedures [43] that, in principle, can be further applied for the sorting of large batches of organisms (e.g., fish, krill, or sea fruits), especially those which accumulate certain elements [44,45,46,47]. Sometimes, the very fact of sample clustering may bring valuable information about processes and their influence on a living organism, such as in studying the reaction of plants to different types of stress [48].

There is also a trend towards combining multiple measurement techniques. In order to use signals of different origin, data fusion techniques such as consensus PCA (CPCA [49]) or common components and specific weights analysis (CCSWA, or ComDim [50,51]) should be used. These techniques allow the description of the overall available information in order to extract the common and distinct information coming from different sources [50,52]. ComDim was specially designed and successfully used to analyse signals of different natures (expert ratings, fluorescence, NIR, MIR, NMR, etc.)—A feature which is appreciated in studying organic compound systems, such as in food quality control [51,53,54,55]. 

In [56,57], ComDim was used in combination with linear discriminant analysis to discriminate samples of instant coffee by means of an electronic nose. A more complicated combination of chemometric algorithms, which included PCA, predictive ComDim, parallel factor analysis, multivariate curve resolution, and PLS-DA, was used for the discrimination of vinegars based on their fluorescence, IR, and NMR spectra [58]. Gibbons et al. [59] combined LIBS and Raman spectroscopies, supported by PCA and LDA, to enhance the identification of clay minerals. The fusion of LIBS and UV-Vis absorption spectroscopy data resulted in very high classification accuracies, up to 100%, for the geographical provenance of Greek olive oils (PCA, LDA, and SVM were used) [60]. 

Thus, we can conclude that the fusion of LIBS and molecular spectroscopy data can result in high classification accuracy for organic samples. Advanced data fusion algorithms such as ComDim can further improve the results; at the same time, this technique, to the best of our knowledge, has almost never been used for atomic spectra (except in [61,62]). In the present study, we assess the potential of LIBS and Raman spectroscopy combined with PCA, NMF, and ComDim for the rapid classification of marine zooplankton animals without visual inspection of whole organisms, with an option of a classification based on the analysis of individual tissues, rather than of whole organisms. 

## 2. Materials and Methods

**Samples**. Twenty-nine dried and pelletised zooplankton samples were involved in the study, namely 14 calanoid copepod (*Calanus spp.*) and 11 *Euphausiacea* crustaceans (totalling 25 crustacean samples), 2 arrow worm samples (*Parasagitta elegans*), 1 sea snail (*Limacina helicina*), and 1 mixed herbivorous zooplankton sample. The animals were caught by the research ships of the Shirshov Institute of Oceanology of the RAS during 2014–2017 expeditions in the Arctic seas and in the Black Sea. For details of the sample description and chemical composition, see Tables S1 and S2. The animals were washed with deionized water, dried at 50° C for 12 h, and pelletized under a moderate pressure (d = 8 mm, 20–1000 bar, typically 30 bar). Due to the inhomogeneity of the samples, we distinguished dark, light, and medium-coloured spots on the pellet surfaces. For each of the colours, LIBS and Raman spectroscopy were carried out in at least 3 spots of that colour, i.e., a total of at least 18 spots were interrogated on each pellet. In the case of LIBS, typically 10 laser shots per spot were delivered. After averaging, the final number of spectra was 29 samples × 3 colours × 2 methods = 174.

**Equipment**. Commercial instruments were employed. LIBS was performed using the J200 Tandem LA-LIBS instrument (Applied Spectra, USA; excitation laser: 266 nm Nd:YAG, 20 mJ/pulse, 10 Hz, spot diameter: 200 μm; acquisition delay: 500 ns). As the instrument was equipped with a CCD detector, we obtained time-integrated spectra (integration time ~3 ms). The spectra covered the entire optical range (186–1049 nm, 12 275 points). Spectral resolution depended on the wavelength range and was typically around 3000 (pixel width ~70 pm with the spectral lines’ full width at the half-maximum of 3 pixels). Automatic background correction was performed by the instrument software.

Raman scattering spectra were recorded using the DXR Raman Microscope (Thermo Scientific, USA; 780 nm laser, energy 1–14 mW, spot size 1–2 μm, spectral resolution 4 cm^−1^) in the range of 450–3150 cm^−1^ (2 801 points). We applied different energies depending on the fluorescent background intensity and the resistance of the samples to burning.

The aperture was selected individually for each sample to attain a sufficient signal-to-noise ratio and avoid CCD overflow at the same time. The following apertures were used: 25 and 50 μm slits and 25 and 50 μm pinholes. The acquisition time ranged from 30 s to 5 min. The spectra were instantaneously fluorescence corrected in the instrument software using a 6th order polynomial function.

**Scheme 1:**
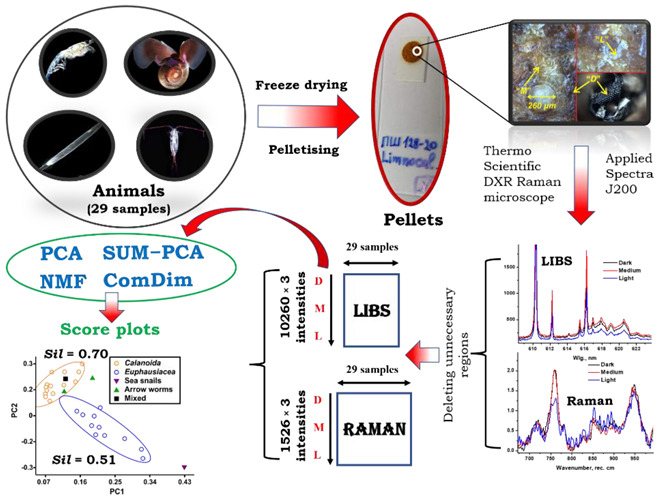
General flowchart of the study.

The general flowchart of this study is shown in Scheme 1. 

**Data processing**. In the emission spectra, the outliers were identified by the Grubbs’ test applied sequentially to the peak intensities of the Li I 610.3 nm, C I 247.8 nm, Na I 568.3 nm, and Ca II 396.8 nm lines and the C_2_ band head at 473.6 nm. After the removal of the outliers, the spectra were averaged for each sample. The Raman spectra were normalised to the total spectral intensity and averaged. The spectra from the spots of different colours were always chained without averaging. Thus, any LIBS or Raman spectrum consisted of three chained spectra.

A number of spectral regions (mainly very intense resonance lines that are strongly self-absorbed in laser plasma [63] and non-characteristic signals) were discarded (see Table S3) to avoid their excessive influence on the decomposition results [62].

A number of matrix decomposition and data fusion algorithms were used for exploratory analysis. Data processing was performed in the GNU Octave, Microsoft Excel, Origin 8.5, Matlab 2020, and Wolfram Mathematica 8 software packages. When necessary, the data tables were divided by their Euclidean norms and again chained (LIBS data–Raman data). In this fashion, concatenated data were obtained (e.g., 29 × 45,228 matrices).

The GNU Octave *princomp* function was used for performing PCA. To determine the right number of principal components (PCs), we used conventional eigenvalue scree plots. This number of PCs was also considered in NMF decompositions (the *nmf_bpas* function). The PCA of concatenated LIBS + Raman datasets is called SUM-PCA. The ICA code made part of a ComDim algorithm (see below) which Emeritus Prof. D. N. Rutledge (AgroParisTech, France) kindly provided to us. In this algorithm, either ICA or PCA could be used for the successive computation of common components (for more details see [62]).

NMF and ICA may be considered as blind source separation (BSS) techniques, which give principal components with a physical sense [35]. The BSS problem is formulated in the following way: determine the N_s_ signals of unknown sources on the basis of the *N_c_* combinations of these signals detected by *N_c_* sensors. If we represent the problem by the relation *x = As*, where *s* and *x* denote the matrices of the source and the observed signals, respectively, and *A* is the so-called mixing matrix, then the goal is to obtain a “de-mixing” matrix *A^−1^* which would convert *x* back to *s* without any a priori information on the sources. The rank of the *A* matrix is determined by the number of sources. The mixing and de-mixing phenomena can be described by different mathematical models; so, different BSS techniques are possible.

In ICA, the statistical independence and the non-Gaussianity of the source vectors are assumed; at the same time, ICA does not imply the orthogonality of the vectors [37,40,41]. Here, we used the ICA implementation based on the joint approximated diagonalization of eigen matrices (JADE) approach, which was a part of the above-mentioned ComDim code.

Conversely, NMF converts a data matrix *X* with *m* rows (descriptors) and *n* columns (samples) into matrices *W* (*m×n*) and *H* (*n×p*), where *p < min (m, n)*. The *p* parameter determines the number of basis vectors and has to be chosen by the user. NMF works so that the resulting matrices will have no negative elements: *X = WH + E*, where *E* is the residual matrix. It is recommended to perform multiple runs of the algorithm with different initial approximations to avoid local minima of *E* [35,36], considering the decomposition with the highest explained variance as the final result of the factorization [64,65].

ComDim (also known as CCSWA) [51,53,54,55] is a method for the analysis of multi-block data. ComDim algorithms determine the latent common dimensions related to each data block and assess the contributions of each of the dimensions to the data blocks. The quantitative measure of this “relevance” is the so-called specific weight, or salience.

If there is a block of measurements *X_k_*, with each of the *N* samples represented there by a line, it is possible to define a cross-product matrix *W_k_ = X_k_×X_k_^T^*. This matrix is then modelled as


$$W_{k}=Q\Lambda^{\left(k\right)}Q^{T}+E_{k}$$

Here, *Q* is an (*N, N*) orthogonal matrix, the columns of which are the common components. The specific weights are stored in *Λ^(k)^*, which is a diagonal (*N, N*) matrix; *Λ^(k)^* can be different in the function of the block *X_k_*, while *Q* is the same for all the data blocks *X_k_*. The sum of the saliences in *Λ*^(k)^ shows the extent to which a particular *X_k_* contributes to *Q*. Saliences can be used to choose the appropriate number of common components, instead of the explained variance values. ComDim calculates the components one by one, and these steps may use PCA or ICA (see above), depending on the actual implementation.

## 3. Results and Discussion

**Figure 1:**
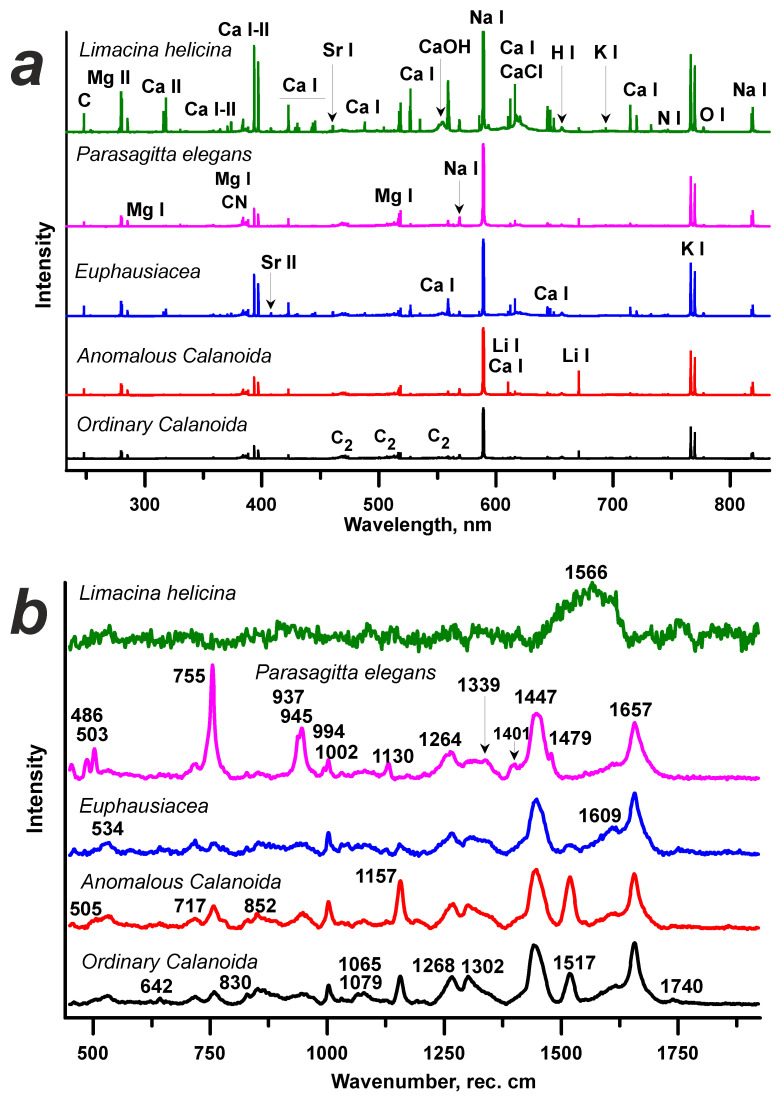
Overview (**a**) LIBS and (**b**) Raman spectra of the studied animals (all from medium-coloured spots). The spectra are averaged over the respective taxa.

The overview spectra of our samples can be seen in Figure 1. Our 29-sample dataset included four different animal taxa (see Table S1). The emission spectrum of the sea snail *Limacina helicina* is the richest in lines because of the presence of a mineralised shell. It is dominated by Ca lines together with strong signals of Na, K, Mg, and molecules such as CaOH and CaCl (Figure 1a). For the reliable identification of atomic emission, we have used the algorithm described elsewhere and earlier [63] (available at https://libs.chem.msu.ru/). The molecular bands were attributed with the help of the Pearse and Gaydon tables [66]. The tissues of arrow worms (*Parasagitta elegans*) are apparently the least mineralised; so, C_2_ and CN molecular emission becomes important in their case. The spectra of *Calanoida* are peculiar for the presence of intense Li lines at 610.4 and 670.8 nm.

Conversely, the Raman spectra of *Limacina* (Figure 1b) are feeble and not very informative. *Parasagitta* present distinct amino acid features such as those at 755 cm^−1^ (probably corresponding to tryptophan) and 945 cm^−1^ (valine), accompanied by sharp peaks at 486, 503, and 3013 cm^−1^ (probably NH_2_ groups). In *Euphausiacea*, relatively strong signals at 1609 cm^−1^ appear (phenyl rings) [67]. *Calanoida* show strong bands of carotenoid pigments at 1004, 1157, and 1518 cm^−1^. Nearly all the animals show strong bands of saturated and unsaturated fatty acids at 1446 and 1656 cm^−1^, respectively [67,68]. These spectral patterns, along with more subtle peculiarities, provide the classification basis. The signals at 1268 and 1302 cm^−1^ are also related to fatty acids.

The performance of chemometric techniques towards the classification of animals was tested using the whole dataset. In addition to visual inspection, we quantitatively assessed the classification performance of the models using the silhouette metric (*Sil*) [2]:

$$Sil=\underset{k}{\sum }\frac{1}{\left|C_{k}\right|}\underset{i\in C_{k}}{\sum }\frac{b_{i}-a_{i}}{\text{max}\left(a_{i},b_{i}\right)},$$

where *a_i_* is the mean distance of a given point *i* from the other points within a cluster *C_k_*, and *b_i_* is the mean distance from *i* to all points outside that cluster. The silhouette values vary from –1 (the worst case, no clusters) through 0 (overlapping clusters) to +1 (distinct compact clusters). In the considerations below, *Sil* will be multiplied by 100 and expressed in % for convenience (Table 1). The *Sil* values were computed for pairs of PCs, which are referred to as “planes” (e.g., a graph in the PC2 vs. PC1 coordinates will be called a “2-1 plane” for brevity). The last column of Table 1 shows multidimensional silhouettes computed using all the relevant subspace. As the relatively subtle discrimination between *Calanoida* and *Euphausiacea* crustaceans turned out to be impossible using the Raman spectra, we computed the Raman silhouettes for the consolidated *Crustaceans* cluster as opposed to the arrow worms and sea snails. Even at that, the silhouette values remained relatively low. Conversely, only *Calanoida* and *Euphausiacea* (a total of 25 samples) clusters were considered for the LIBS and fused spectral data.

**Figure 2:**
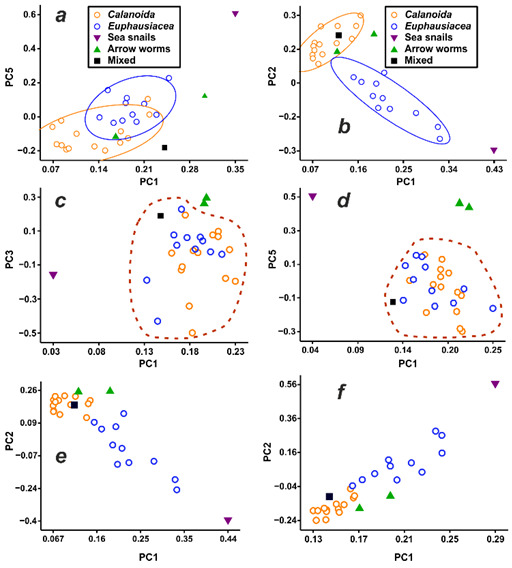
Score graphs for the PCA decompositions of LIBS (**a**,**b**) and Raman (**c**,**d**) spectra before (**a**,**c**) and after (**b**,**d**) masking the strongest signals. Dashed lines enclose the crustacean samples; (**e**) LIBS spectra without Li signals; (**f**) SUM-PCA for LIBS + Raman data without Li signals. In (**a**,**b**), confidence ellipses (*p* = 0.95) for the respective clusters are shown.

First, note how different the silhouettes are for the raw and shortened spectra (i.e., with and without resonance lines—see the experiment section): 10 vs. 65% and 34 vs. 62% for the Raman and LIBS data, respectively (Figure 2a–d). For the LIBS, the “best plane” with the largest silhouette also shifts back from 5-1 to 2-1 due to the avoidance of the excessive influence of the resonance lines on the explained variance (in the 2-1 projection, the classification is driven by the C_2_ and Li emission). Moreover, the confidence ellipses, which overlap in Figure 2a, become distinct in Figure 2b. Thus, in what follows we will imply the shortened datasets only.

**Figure 3:**
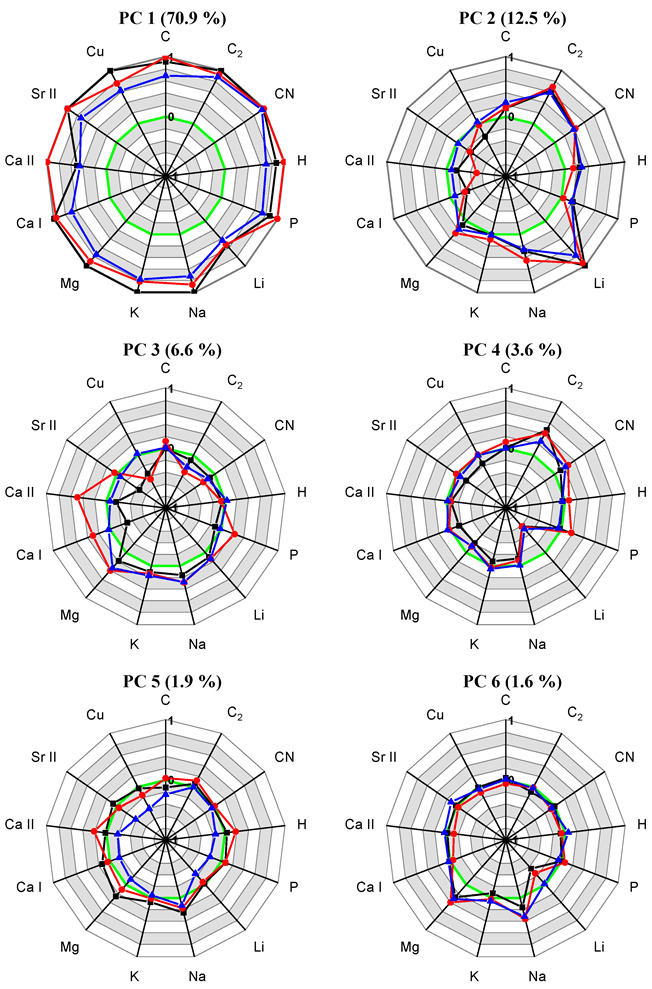
Selected features of PCA loadings for LIBS. Black, red, and blue lines correspond to dark, medium-coloured, and light spots, respectively. The width of the rings is 0.2. The green circle is at the zero level.

As for the LIBS data, the PCA loadings are crowded with signals, most of which are correlated; it is convenient to choose a limited number of representative signals and show them on spider diagrams such as those in Figure 3. Here, the integrals of selected peaks of C, C_2_, CN, H, P, Li, Na, K, Mg, Ca I, Ca II, Sr II, and Cu are shown (see details in Table S4); each one is normalized to the respective greatest absolute magnitudes among all the principal components.

Surprisingly, the discrimination power of PCA with the Raman data is much worse than with the LIBS data. The cause of this may be the relatively low variability (and limited number) of the Raman signals. The PCA for the Raman data resulted in the separation of carotenoid and amino acid (tryptophan and valine) signals between different components (not shown). The best score graph is for the 5-1 plane (Figure 2d), which only involves the amino acid-related PC No. 5.

Emission spectra contain many sharp, distinct, and highly variable spectral features; this results in a better discriminative power. The PCA of the LIBS data yields two loadings (nos. 2 and 4 in Figure 3) with a prominent Li signal (with almost no other signals), which may suggest two different forms of Li in the tissues or two different accumulation patterns. PC1 apparently represents a basic composition which is common to all the samples. PC3 and PC5 highlight the importance of Ca I and Ca II emission in some samples. PC6 shows a correlation between magnesium and sodium. The loadings for the dark, medium-coloured, and light spots are obviously correlated to a high extent. One might think that the discrimination in Figure 2b is driven by the anomalously high Li content, which is characteristic of *Calanoida* [44,45]. It is, however, easy to see that the *Parasagitta* worms, which do not contain any lithium, fall close to *Calanoida* on the score graphs. Moreover, the masking of Li signals does not significantly alter the results of the decompositions. It is, rather, the calcium and strontium signals which drive the classification. Ca and P outbreaks in the PC3 can indicate the presence of calcium phosphate in the medium-coloured material.

**Figure 4:**
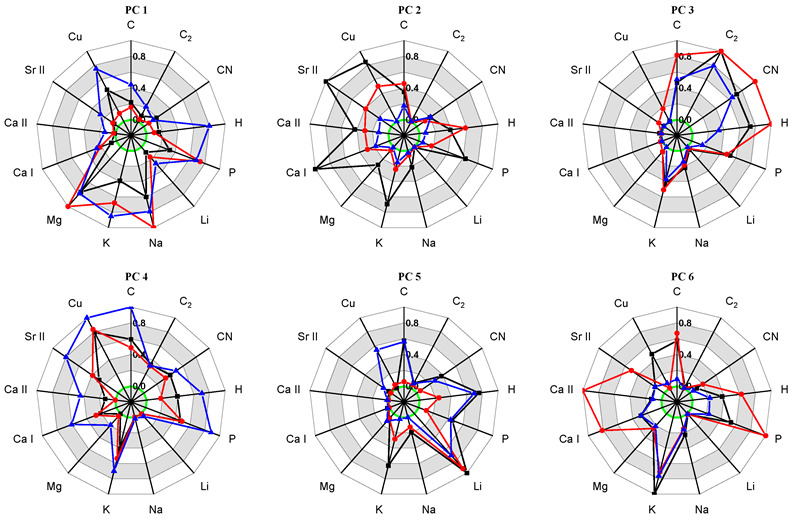
NMF decomposition of LIB spectra (without resonance lines). Shown are integrals of selected peaks on graphs of loadings, normalized to respective greatest absolute magnitudes among all the principal components. For wavelength positions of the peaks, please see the main text and supplementary tables. Black, red, and blue lines correspond to dark, medium-coloured, and light spots, respectively. The width of grey and white rings is 0.2.

To get a deeper insight into the nature of the signals, we used NMF since it is likely to find physically meaningful components (e.g., signal sets related to a chemical compound or a group of compounds) [35,65]. We found that NMF loadings of the LIBS data do have different shapes for light, medium-coloured, and dark spots (Figure 4). The cause of this may be that the features cannot be easily smoothened by the factorization algorithm because of the non-negativity constraint.

The resonance line masking was quite efficient in improving the NMF-related silhouettes (see Table 1 and Figure 4). The Li signal is present together with K in one of the components and with Na, Mg, and K in another. It is noteworthy that the loadings for the dark, medium-coloured, and light spots are not so strongly correlated as in the PCA. Potassium occupies a special place, being present in all components and correlated with several groups of elements. As in the PCA, there is co-presence of Mg and Na in one of the components (PC1). The relation of Ca and P in PC6 is reminiscent of the phosphate minerals which can be present in the exoskeletons of crustaceans (this corroborates the above-discussed PCA results). As NMF gives non-orthogonal components, the respective score graphs are constructed in scalene rather than in rectangular coordinates. The inter-axis angle, *α*, is given by the relation: $\cos\alpha =\left(u,v\right)/\|u\|\|v\|$, where *u* and *v* are any two of the latent vectors obtained as a result of a decomposition. By applying standard trigonometry, it is possible to plot NMF scores (the H matrix) in these scalene coordinates.

**Figure 5:**
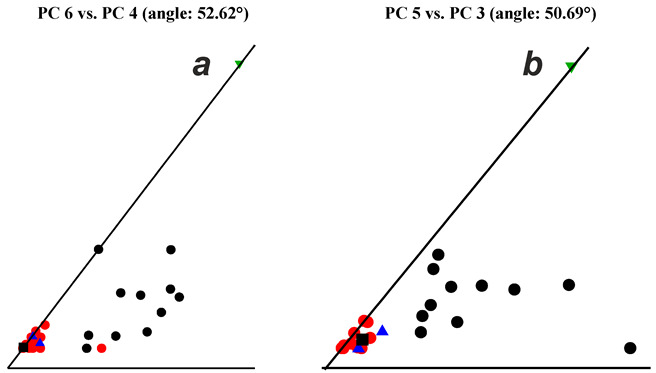
Score graphs of NMF decompositions: (**a**) LIBS only (cf. Figure 4); (**b**) fused Raman + LIBS dataset. Shown are projections with best silhouette metrics.

The graph in Figure 5a shows a relatively good discrimination between *Calanoida* and *Euphausiacea*. It is again noteworthy that Li plays no role in this since the relevant latent vectors (nos. 6 and 4, cf. Figure 4) do not contain its signals. The difference between these vectors lies in the intensities of the Ca, Sr, Na, Cu and C_2_ signals. For the *Euphausiacea* samples, high scores in both directions are observed, in contrast to *Calanoida* and *Parasagitta*. This just means that the latter are less mineralised. One of the *Calanoida* samples (No. 29) falls into the wrong cluster due to its anomalously high Sr content, which drives the point far to the right along the PC4 axis. This error, however, disappears when Raman data are also considered (NMF: see Figure 5b).

**Figure 6:**
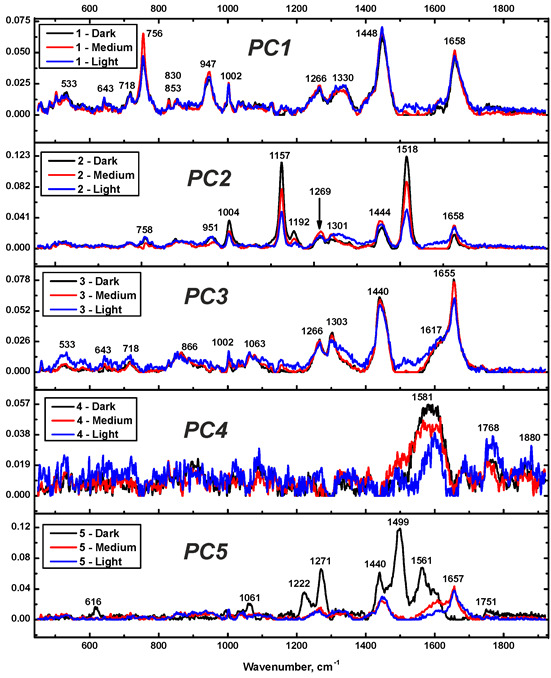
NMF decomposition of Raman spectra. Loadings obtained from spectra spanning the range of 449–1921 cm^−1^. Black, red, and blue lines correspond to dark, medium-coloured, and light spots, respectively.

As with the PCA, the NMF factorization of the Raman spectra (Figure 6) did not provide a good sample clustering (the classification seems to be driven mainly by carotenoid bands). Carotenoid compounds and amino acids appear in two different components, though they are not free from the fatty acid bands, which themselves appear in a separate component. At the same time, the intensity of the carotenoid bands differs greatly as a function of spot colour, which is probably because these compounds are responsible for the tissue colour. PC4 looks quite like the *Limacina helicina* spectrum (Figure 1b). The explained variance is lower in the NMF than in the PCA for same datasets (see Table 1).

**Figure 7:**
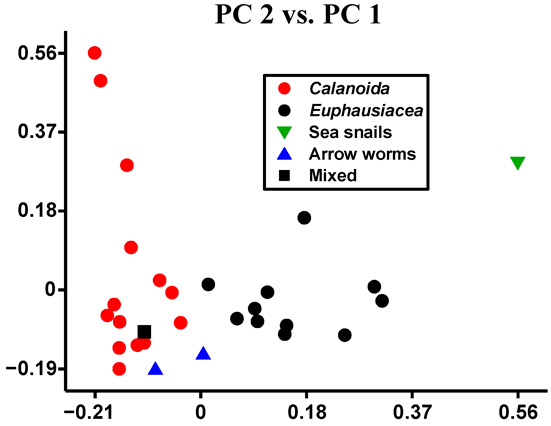
The best score graph for the ComDim–PCA decomposition.

ComDim was tested in two variants: PCA-based and ICA-based. For both of them, the explained variance (EV) was very high. Despite the fact that the obtained loadings were easy to interpret and thus instrumental for finding correlations between the chemical components, the respective score graphs did not yield well-defined clusters, and the corresponding silhouette values were relatively modest. The best score graph (for ComDim–PCA) is shown in Figure 7 as an example.

Table 1 shows that the LIBS data were always modelled with higher explained variance (EV) than the Raman data. Although NMF yielded a much lower EV, its performance in discrimination was only slightly inferior to that of PCA. The SUM-PCA for the LIBS + Raman data was the best for the discrimination. Additionally, there was apparently no clustering of the *Calanoida* samples according to their geographical provenance (the Arctic Ocean or the Black Sea). This can be considered both as a strength and a limitation of the used protocol. On the one hand, it is not possible to distinguish the animals coming from the different regions, but on the other hand, this very fact makes the order-level discrimination more robust.

### Critical Analysis and Discussion

In addition to visual inspection and computer vision [69], hyperspectral imaging [70] was also implemented for zooplankton species classification [71], but it is necessary to have an intact organism for reliable results. Conversely, LIBS and Raman spectroscopy can be adopted for microanalytical measurements, because they can deal both with individual millimetre-sized planktonic organisms and their parts (damaged bodies, etc.) since the achievable spatial resolution of LIBS and Raman is down to 5–10 µm [72,73]. In all cases, however, we should initially evaluate the classification performance of both techniques towards particular samples—this is why we have focused on pelleted samples and a relatively large sampling area in the current study.

Although the clusterisation quality obtained by us is better than in earlier studies [74,75], it would have been remiss to not investigate the capabilities of the chemometric techniques (PCA, NMF, and ComDim) in finding the latent characteristic features in the spectra of animals. Similar works do exist [5,76], but they usually either do not go further than conducting PCA or, on the contrary, use complicated algorithms requiring big training datasets (such as neural networks). We believe we have emphasized the fitness of NMF and ComDim for the classification and discrimination purposes and have also shown that PCA is still competitive, despite the existence of more sophisticated techniques.

## 4. Conclusions

We have tested the performance of several chemometric techniques (PCA, NMF, ComDim–PCA, and ComDim–ICA) for the unsupervised classification of zooplankton species based on their LIBS and Raman spectra. Given that these algorithms and the silhouette metric are rarely applied in analytical studies, we have emphasized their fitness for classification and discrimination purposes; this fitness is based on their capabilities in finding latent characteristic features in the spectra of animals. The LIBS data were substantially more useful for this task than the Raman spectra, although the best results were achieved for the combined LIBS + Raman dataset. Although NMF and ComDim gave interesting information in the loadings, the common SUM-PCA is the best solution for the discrimination based on the score graphs. It was not difficult to distinguish between the *Calanoida* and *Euphausiacea* crustaceans and the *Limacina helicina* sea snails because of their different mineral compositions. Conversely, the arrow worms (*Parasagitta elegans*) usually fell into the same class with *Calanoida* despite the differences in their Raman spectra. This issue could probably be solved in the future by using weighted mathematical algorithms. Apparently, the geographical provenance of the samples did not influence the results, which suggests that the found features belong to all the animals of the respective species, not only to a specific local population.

The results of the present study may be used for the automated LIBS + Raman-based sorting of large batches of organisms (e.g., fish, krill, or sea fruits). However, the most important result is that it has been possible to successfully perform a classification of animals from the presented set of samples because their chemical compositions turned out to be really different from each other. Further experiments with more samples and more species can provide a more detailed vision of the interrelations in the chemical compositions of sea creatures.

## Figures and Tables

**Table 1:** Silhouettes (in %) for all the score graphs involved in the present study.

No.	Method, Dataset	Plane	*Sil*, %	*Sil (all dim.),* %
Crustaceans–Arrow worms–Sea snails discrimination				
1	PCA—Raman only, full (5/97)	3-1	10	–10
2	PCA—Raman only, short (5/89)	5-1	65	39
3	NMF—Raman only, full (5/84)	4-2	11.5	–6
4	NMF—Raman only, short (5/75)	4-1	47	34
*Calanoida*–*Euphausiacea* discrimination				
5	PCA—LIBS only, full (5/99)	5-1	34	17
6	PCA—LIBS only, short (6/97)	2-1	62	22
7	NMF—LIBS only, full (5/88)	5-2	56	6
8	NMF—LIBS only, short (6/83)	6-4	60	37
9	SUM-PCA—LIBS + Raman (5/91)	2-1	67	21
10	NMF—LIBS + Raman (5/72)	5-3	63	28
11	ComDimPCA—LIBS + Raman (3/99.06)	2-1	39	30
12	ComDimICA—LIBS + Raman (3/96.89)	3-2	36	25

“Full” refers to intact spectra; “short” means that some signals were masked as described in the experiment section. The “Sil, %” column shows the maximum silhouette values among all the available 2D score graphs. Planes in which maximum values are obtained are indicated in the second column (e.g., 5-1 means PC5 vs. PC1 plane). Notations such as (5/99) mean that there are 5 PCs explaining 99% of variance. The “Sil (all dim.), %” column shows the multi-dimensional silhouette values computed using the whole available subspace.

## Data Availability

The data presented in this study are available in the Supplementary Materials.

## Supplementary Materials

The following supporting information can be downloaded at: https://www.mdpi.com/article/10.3390/s22218234/s1, Table S1: collection conditions of zooplankton samples, Table S2: the results of elemental analysis of zooplankton by ICP-AES and ICP-MS, Table S3: list of spectral ranges eliminated from LIBS data to improve the performance of chemometric algorithms, Table S4: list of emission signals used to construct spider diagrams.
